# Bioactive Plant Metabolites in the Management of Non-Communicable Metabolic Diseases: Looking at Opportunities beyond the Horizon

**DOI:** 10.3390/metabo5040733

**Published:** 2015-12-12

**Authors:** Chandan Prasad, Victorine Imrhan, Shanil Juma, Mindy Maziarz, Anand Prasad, Casey Tiernan, Parakat Vijayagopal

**Affiliations:** 1 Department of Nutrition and Food Sciences, Texas Woman’s University, Denton, TX 76204, USA; E-Mails: Vimrhan@twu.edu (V.I.); sjuma@twu.edu (S.J.); CTiernan@twu.edu (C.T.); pvijayagopal@twu.edu (P.V.); 2 Department of Medicine, LSU Health Sciences Center, New Orleans, LA 70112, USA; 3 Department of Nutrition and Food Sciences, Texas Woman’s University, Houston, TX 77030, USA; E-Mail: Mmaziarz@twu.edu (M.M.); 4 Department of Medicine, Division of Cardiology, UT Health Science Center at San Antonio, 7703 Floyd Curl Drive, San Antonio, TX 78229, USA; E-Mail: Prasada@uthscsa.edu (A.P.)

**Keywords:** cardiovascular disease, diabetes, food bioactives, functional food, resistant starch, polyphenols, cyclo (His-Pro)

## Abstract

There has been an unprecedented worldwide rise in non-communicable metabolic diseases (NCDs), particularly cardiovascular diseases (CVD) and diabetes. While modern pharmacotherapy has decreased the mortality in the existing population, it has failed to stem the rise. Furthermore, a large segment of the world population cannot afford expensive pharmacotherapy. Therefore, there is an urgent need for inexpensive preventive measures to control the rise in CVD and diabetes and associated co-morbidities. The purpose of this review is to explore the role of food bioactives in prevention of NCDs. To this end, we have critically analyzed the possible utility of three classes of food bioactives: (a) resistant starch, a metabolically resistant carbohydrate known to favorably modulate insulin secretion and glucose metabolism; (b) cyclo (His-Pro), a food-derived cyclic dipeptides; and (c) polyphenol-rich berries. Finally, we have also briefly outlined the strategies needed to prepare these food-bioactives for human use.

## 1. Introduction

We have made a remarkable progress in public health and healthcare, including managing communicable diseases in the last 100–150 years. Some of the notable human achievements include discovery of antibiotics, starting with first antibiotic by Flemming in 1930s [1], vaccines [2], clean water supplies and better sewage disposal and, finally, fortification of foods to combat micronutrient deficiencies in many parts of the world [3]. These have led to a significant increase in life expectancy throughout the world. For example, as per UN estimates life expectancy between 2000 and 2050 is projected to increase by 16 years even in some of the poorest nations of Africa [4]. This is the good news. However, something else has happened with the status of the health of world citizens that is perhaps more alarming. There is a steady upsurge in non-communicable metabolic diseases (NCDs), diabetes, cardiovascular disease (CVD), obesity, and others worldwide since the 1970s, if not earlier. For example, as per United Nations estimates, the incidence of diabetes and CVD in China and India, the two most populous nations of the world representing about one-third of the world population, is projected to double with a total number exceeding 100 million by year 2025 [5].

The reasons underlying this remarkable change in human health is complex and perhaps has a lot to do with our lifestyle than genetics. Therefore, it is imperative that we have a discussion on how our lifestyles have changed over the last decades. The period following WWII has seen relative peace, agricultural revolution in 1970s that brought significant reduction in world food shortages, and food fortification with micronutrients such as vitamins and minerals. While this brought better food security worldwide, there were other more recent changes that possibly may have impacted world lifestyle, hence health. Some of these include wide-spread imbalance in current dietary choices, overuse of refined hyper-processed foods, and low intake of fibers and plant-derived bioactives. All such factors are beyond the scope of this review. However, it would be appropriate to enumerate some of these factors that have been reviewed in detail elsewhere. Some of these include, global changes in income distribution [6], globalization leading to change in eating habits [7,8,9], rural migration and urbanization [10,11], and automobiles, television, and inactive entertainment instruments [12,13].

Based on extensive animal studies and human epidemiologic studies, we are certain that obesity plays a central role in the etiology of diabetes and CVD. Therefore, the major focus of this review will be to examine whether nutrition via delivery of functional foods or food bioactives could help reduce incidence of obesity, a precursor to two major NCDs—diabetes and CVD. Using management of dyslipidemia as an example, we have next summarized successes and failures of current use of statin therapy, as well as alternatives to statins.

## 2. Successes and Failures of Current Strategies for Management of Non-Communicable Diseases: Lipid Lowering Therapy as an Example

The use of HMG-COA reductase inhibitors (statins) has grown in frequency and intensity over the past two decades for secondary reduction of cardiovascular events and for the primary prevention of events in high risk individuals. The most recent iteration of the blood cholesterol guidelines put forth through the collaboration of the American College of Cardiology, the American Heart Association, and the National Heart Lung and Blood Institute have been met with controversy. These agencies have jointly suggested use of fixed dose, high- or moderate-intensity statin-based lipid-lowering strategies based upon the patient’s risk profile rather than targeting specific low density lipoprotein (LDL) or high density lipoprotein (HDL) goals [14]. Evidence would suggest that the latest iteration of these guidelines is projected to increase the number of patients treated with statin therapy [15]. Along with this increased utilization will be the ever-present concern of “statin intolerance”. This review will outline the therapeutic strategies based on both naturally occurring and man-made pharmaceutical agents to consider in statin intolerant patients.

### 2.1. Statin Intolerance

Statin intolerance is most commonly defined as the inability to continue statin therapy or tolerate increased intensity of therapy due to side effects with or without blood evidence of muscle or liver abnormalities. Although, the incidence of frank liver failure, or rhabdomyolysis, is exceedingly rare (less than 1 in 20 million), the development of milder symptoms of myalgias and fatigue are more common and is estimated to be present in 10%–18% of patients [16]. Muscular side effects remain a major source of statin intolerance and reason for discontinuation of therapy. Advanced age, female gender, low muscle mass, baseline liver disease, and concomitant use of gemfibrozil all increase the risk of developing statin intolerance [17,18]. Multiple different dosing strategies have been recommended to manage statin intolerance while maintaining therapy including: use of lower dosages, intermittent dosing, administration of slow release agents (fluvastatin), trial of alternative statins, and minimization of drug-drug interactions [18].

### 2.2. Coenzyme Q10

Although the complete etiology of the myalgias remains unclear, the reduction in coenzyme Q10 (CQ10) in myocytes has been implicated as a potential source for this adverse effect [19]. Empirically, the use of CQ10 supplementation has been used in patients with varying degrees of success. A meta-analysis of placebo-controlled trials of statin use confirmed a significant reduction in serum levels of CQ10 with statin therapy [20]. Unfortunately, there has not been consistent evidence from randomized data to support supplementation with CQ10 in patients with myopathy [20]. A recent systematic review supports this clinical equipoise and, given the low risk of side effects of CQ10, suggests that individual patients may derive benefit—in part due to placebo effect [19].

### 2.3. Vitamin D Supplementation

Apart from CQ10, recent focus has turned to vitamin D levels as an important factor in the development of statin related myalgias. Using data from the National Health and Nutrition Examination Survey 2001–2004, Morioka *et al.*, demonstrated that middle aged and older adult statin users with serum 25-hydroxyvitamin D (25[OH]D) levels <15 ng/mL, had nearly two times greater odds of reporting musculoskeletal pain compared to non-statin users [21]. Whether this association is causative or not is unclear and at this time speculative hypotheses note that vitamin D deficiency can lead to atrophy of type II muscle fibers—the clinical manifestations of which could be magnified by statin use. A more recent prospective clinical study in statin intolerant patients with vitamin D levels <32 ng/mL, demonstrated that with sufficient vitamin D supplementation (50,000–100,000 units/week), up to 95% of patients could be maintained on statin therapy without symptoms or myonecrosis and this re-initiation of lipid-lowering therapy was associated with an improved cholesterol profile [22]. At this point, use of vitamin D remains attractive and may be an option in deficient patients, although large randomized studies and mechanistic data remain scarce.

### 2.4. Alternatives to Statins

In individuals who are truly intolerant to statin therapy, alternative agents may be considered. The common themes surrounding these therapies is a lower intensity of LDL lowering, relatively fewer cardiovascular outcomes data, and additional side effects that may complicate tolerability. The agents available in this context include vitamin B3 (niacin or nicotinic acid), fibrates (amphipathic carboxylic acids), cholestyramine (bile acid sequestrants), plant sterol esters, red rice yeast, eztimibe, and the newly approved proprotein convertase subtilisin/kexin type 9 (PCSK9) inhibitors. Although a full discussion of the data for each of these agents is beyond the scope of the present review, Table 1 summarizes the key clinical and pharmacological points.

### 2.5. PCSK9 Inhibition: The Future?

There is considerable interest in a newly approved class of medications, the PCSK9 inhibitors. These agents include evolocumab, alirocumab, and bococizumab, and are human monoclonal antibodies against PCSK9—administered as a subcutaneous injection every two weeks [23]. PCSK9 is a serine protease which binds to the LDL receptor leading to the intra-hepatocyte degradation of the receptor [24]. The effect of these drugs is to increase LDL receptor expression in the liver and facilitate LDL clearance—thereby lowering serum LDL cholesterol. While clearly an attractive treatment for patients with familial hypercholesterolemia who have mutations of the PCSK9 gene and are difficult to treat with statins, the potential of these agents lies in treatment of the larger population of dyslipidemic patients. The various agents appear to have a class effect in terms of LDL lowering with comparable magnitude of effect. The first agent to receive United States Food and Drug Administration (FDA) approval was alirocumab (July 2015) and, per the FDA, approval is indicated for additive therapy to dietary modification and maximally tolerated statin therapy in adult patients with heterozygous familial hypercholesterolemia or patients with clinical atherosclerotic cardiovascular disease such as heart attacks or strokes, requiring additional lowering of LDL cholesterol. Interestingly, dose escalation of statins up-regulates PCSK9 which explains the magnitude of LDL lowering. PCSK9 monotherapy has been shown to reduce LDL levels from 40% to over 50% [25]. When coupled with a statin (even at a low dose) the reductions range from 40% to 70%. The use of PCSK9 inhibitors in statin intolerant patients is particularly attractive given the relatively mild side effect profile. The GAUSS-2 (Goal Achievement after Utilizing an Anti-PCSK9 Antibody in Statin Intolerant Subjects) study examined the relative effect of LDL lowering by evolocumab compared to ezetimbide in patients who were statin-intolerant due to muscle-related side effects [26]. The LDL lowering of evolocumab in the study was 53%–56% as compared to 15%–18% with ezetimibe.

**Table 1:** Agents commonly used for the management of dyslipidemia.

Agent/Dose	Mechanism of Action	Effects on Lipid Profile	Common Side Effects	Key Clinical Data
Niacin	Inhibits triglyceride (TG) synthesis and favorably impacts apoliprotein B containing lipoproteins	Two gram dose can lower low density liproprotein (LDL) by 15%, increase HDL by up to 25%, and lower TG by up to 30%	Flushing, gastrointestinal effects, pruritis, and rash	Randomized controlled trials of niacin including AIM-HIGH and HPS2-THRIVE have demonstrated no benefit in cardiovascular events despite significant increases in HDL levels [27,28].
Fibrates	Reduces hepatic TG production, enhances LDL uptake by the LDL receptor, and stimulates lipoprotein lipolysis	Primarily lowers TG and can lower LDL by ~10%	Gastrointestinal side effects including nausea and diarrhea. Can raise liver enzymes and cause gallstone formation. Drug-drug interactions with statins and warfarin are important to watch	Meta-analyses have demonstrated a reduction in coronary events including non-fatal MI. However, no benefit in all-cause mortality was seen [27,28]
Bile acid sequestrants	Bind to bile acids in the intestine and prevent recirculation of cholesterol	Depending on dosage, can decrease LDL 15%–30%	Gastrointestinal side effects including constipation, very rare reports of myalgias	May have beneficial impact on coronary atherosclerosis and may lower HbA1c [27].
Plant sterol esters	Interfere with dietary and biliary cholesterol absorption from the intestines	2–3 grams per day may reduce LDL cholesterol by 8%–15% (typically 8%–9%) and TG by 6%–9%	Well tolerated, uncertain impact on vitamin and nutrient absorption Uncertain impact on cancer risk	Limited data on markers of atherosclerosis and no large randomized cardiovascular outcomes data [29].
Red rice yeast	Functionally a low dose statin. Contains monacolin—the active ingredient in lovastatin is monacolin K	Reductions in LDL vary between 19% and 30% Minimal impact on HDL and modest effect on TG reduction	Generally well tolerated—even in statin intolerant patients but there have been reported myalgias similar to statins with one case of rhabdomyolysis reported.	Several small randomized controlled trials. Recent meta-analysis of 13 trials confirms LDL reduction, modest TG reductions and no impact on HDL levels [30]. No significant creatinine kinase level changes were noted.
Ezetimibe	Decreases cholesterol absorption in the small intestine	As an alternative to statin therapy can lower LDL cholesterol ~15%–20%. Minimal impact on HDL as monotherapy. With statin use can lower LDL by ~25%–60% with moderate reduction in TG. Can be used with fibrates to achieve ~20% lowering of LDL, and moderate increase in HDL, and decrease in TG	Generally well tolerated. Most common side effects: diarrhea (2.0%–2.5%), myalgias with statin use (3%), transaminitis with statin use (1.3%)	Clinical efficacy and safety for LDL lowering have been established in multiple studies. IMPROVE-IT randomized 18,144 recent acute coronary syndrome patients to simvastatin + ezetimibe *vs.* simvastatin + placebo [31]. There was a median follow up of six years and the primary composite endpoint was death from CVD, a major coronary event (nonfatal myocardial infarction (MI), documented unstable angina requiring hospital admission, or coronary revascularization occurring at least 30 days after randomization), or nonfatal stroke. The trial demonstrated an absolute risk reduction of 2.0% (*p* = 0.016) in the primary endpoint, driven by a significant reduction in non-fatal MI and stroke.

These data suggest a role of this new class of drug as monotherapy for LDL lowering in statin intolerant individuals. The missing link is in PCSK9 inhibition therapy is the impact of these drugs on cardiovascular outcomes. Nearly 60,000 high-risk patients are being evaluated in randomized controlled trials to address this question. In addition, long term safety data and elucidation of any pleiotropic effects remains to be determined.

Based on over three decades of study, the LDL hypothesis as a central feature of atherosclerosis and adverse cardiovascular outcomes remains the focus of lipid management. Statin intolerance, though less common in randomized trials, is not uncommon in clinical practice. The majority of non-statin LDL-lowering drugs have modest LDL reductions and have additional side effects. The novel class of PCSK9 inhibitors appears to offer a new option for statin intolerant patients with profound reduction in serum LDL cholesterol without myalgias. The long term efficacy and safety of these drugs requires further evaluation. The next section presents a general overview food bioactives that may have a role to play in the management of CVD and diabetes.

## 3. A Brief Survey of Foods Rich in Bioactive Plant Metabolites

The relationship between diet and chronic diseases has been extensively studied. For many years research focused on reducing total fat, saturated fat, cholesterol, and trans-fat in the diet to reduce the risk of certain chronic diseases, such as CVD and stroke. However, dietary modifications that include plant products have been shown to reduce the risk of these chronic diseases. The Nurses’ Health Study, the Health Professionals’ Follow-up Study [32,33], Women’s Health Study [34], and the Physicians’ Health Study [35] show that increasing consumption of fruits and vegetables resulted in decreased risk for CVD. Similarly, Bazzano *et al.* [36] reported that consumption of fruits and vegetables was inversely associated with stroke incidence, stroke mortality, ischemic heart disease mortality, and CVD mortality. In addition to fruits and vegetables, grains have been reported to reduce the risk of CVD [37] and of all-cause mortality in postmenopausal women [38]. Known modifiable risk factors for CVD include smoking, sedentary lifestyle, diet, dyslipidemia, hypertension, obesity, and type 2 diabetes [39].

The observed protective effect of consuming plant foods on chronic diseases is likely due to their bioactive components. The plant bioactive compounds that will be discussed in this review include phytosterols, flavonoids, and phytoestrogens (lignans). We will focus on effects of these bioactive compounds on CVD and type 2 diabetes.

### 3.1. Phytosterols

Phytosterols are naturally-occurring plant sterols found in the non-saponifiable fraction of plant oils. Plants synthesize several types of phytosterols (e.g., sterols and stanols) that are structurally similar to cholesterol, except for the functional group substitutions on the sterol side chain at the C24 position. Both cholesterol [40] and phytosterols [41,42] are important components of cell membranes responsible for regulating membrane fluidity and permeability.

Of all the phytosterols that have been identified, beta-sitosterol (most abundant), campesterol, and stigmasterol comprise almost our entire intake of phytosterols. Since humans do not synthesize phytosterols, they must be obtained from the diet. The main dietary sources of naturally-occurring phytosterols are vegetable oils, nuts, grains and, to a lesser extent, fruits and vegetables [43]. Phytosterols intake has been reported to decrease blood cholesterol despite their poor absorption (<10%) from the intestine [44]. In the intestine, phytosterols displace cholesterol in mixed micelles and, thus, inhibit the absorption of cholesterol [45] reducing circulating LDL concentration [46,47], a known risk factor for CVD.

As a result of the reported health benefits of phytosterols, and the small amounts that occur naturally in foods, FDA in 2000 authorized the use of a health claim—“may reduce the risk of heart disease” for foods containing phytosterols (beta-sitosterol, campesterol, and stigmasterol). For such a claim to be made, the food must contain “at least 0.65 g per serving of plant sterol esters or 1.7 g per serving of plant stanol esters, and consumed twice a day with meals for a daily total intake of at least 1.3 g of sterol esters or at least 3.4 g of stanol esters, as a part of a diet low in saturated fat and cholesterol may decrease the risk of heart disease [48]. In response to health petitions received, the FDA in 2010 proposed new requirements for a claim to be made for fortification of foods with phytosterols. “Foods containing at least 0.5 g per serving of plant sterols/stanols (or plant sterols and stanols) eaten with meals or snacks for a daily total intake of 2 g as a part of a diet low in saturated fat and cholesterol, may reduce the risk of heart disease [49].

The food industry seized the opportunity to provide commonly consumed products that are fortified with phytosterols, such as Benecol™ and Take Control™. Benecol spread contains stanol esters derived from tall oil (pine tree wood pulp) and Take Control margarine contains sterol esters from soybeans. Consuming 2–3 g/d of phytosterols from these products resulted in approximately 14% reduction in LDL with no change in HDL [50,51,52]. Thus, both sterols and stanols are equally effective in lowering LDL concentration. In addition to margarines and spreads, a variety of food groups (grains, cereals, dairy products, and salad dressings) were fortified with various amounts of stanols and sterols. Doses as low as 0.8–1.0 gram/day resulted in approximately 5% reduction in LDL concentration with higher doses (2 g/d) having greater cholesterol-lowering effect (~10%) [53,54,55]. The cholesterol-lowering effect of phytosterols has been shown to last up to one year [56,57]. These observations have resulted in the recommendation from the National Cholesterol Education Program (NCEP) Adult Treatment Panel III to include two grams of plant sterol or stanol esters daily for optimal dietary therapy for elevated LDL [58]. Recently, guidelines for lowering LDL concentration issued by the American Heart Association include consumption of a diet that emphasizes vegetables, fruits, whole grains, legumes, and nuts [38], which are foods that contain naturally-occurring phytosterols.

### 3.2. Flavonoids

This group of polyphenols is the most abundant in the human diet. It can be classified into many different categories based on their structures. The most common flavonoids are flavones, flavanols, catechins, and anthocyanins, along with anthoxanthins [59,60]. Epidemiological studies show an inverse relationship between flavonoid intake and chronic diseases including CVD [61,62,63,64]. When compared to low (<19 mg/d) intakes of flavonoids, individuals with higher intake (30 mg/d) had approximately 50% reduction in CHD mortality [64]. Similar reductions were observed with the consumption of apples [62] or onions [61]. The protective effects of flavonoids can be linked to the antioxidant capacity of these compounds and their metabolites.

Red wines contain an abundance of polyphenols including phenolic acids (for example, gallic acid, and caffeic acid), stilbenes (resveratrol), and flavonoids (for example, catechin, epicatechin, quercetin, rutin) [65]. The most abundant polyphenol in red wine is caffeic acid, but gallic acid had the highest antioxidant activity [66]. The polyphenols found in red wine inhibit oxidation of LDL *in vitro* [67,68] and increase total antioxidant capacity in the serum [69]. The polyphenols in red wine have antithrombotic effects due to decreased platelet aggregation, reduced synthesis of pro-thrombotic and pro-inflammatory mediators, and decreased expression of adhesion molecules [70,71]. In addition, red wine polyphenols can induce vasorelaxation via nitric oxide synthesis [71]. The protective effect of red wine polyphenols is independent of the alcohol content. Freedman *et al.* [72] reported that purple grape juice (7 mL/kg body weight per day for 14 days) decreased platelet aggregation (58% *vs.* 39%), increased nitric oxide release, and suppressed superoxide production. According to Xiang *et al.* [73] resveratrol exhibited lower antioxidant activity than gallic, caffeic, and syringic acids. However, the addition of resveratrol to the wine samples did not significantly improve the antioxidant activity, suggesting that resveratrol may exert its health effects by other mechanisms [73].

Resveratrol is a polyphenol found principally in the skin of grapes and, in lesser amounts, in peanuts. Moderate consumption of red wine (French paradox) reduced the risk for CVD via several mechanisms. Unlike observation of Xiang *et al.* [73], others have shown resveratrol to effectively scavenge free radicals and other oxidants [74,75]. Resveratrol inhibits both LDL oxidation [63] and platelet aggregation [76], as well as cyclooxygenase-2 (COX-2) synthesis [77]. Platelet aggregation is one of the first steps in blood clot formation. Blood clots have been known to occlude arteries resulting in myocardial infarction or stroke. One of the earliest steps in the development of atherosclerosis is recruitment of inflammatory cells from the blood to the arterial wall by vascular cell adhesion molecules (VCAM), and *in vitro* studies show resveratrol decreased the expression VCAM-1 [78] and matrix metalloproteinase-9 (MMP-9) mRNA [79] by suppressing nuclear factor AP-1 activation [77]. Resveratrol may also inhibit pro-inflammatory transcription factors such as NFκB or AP-1 [80]. The cardioprotective effect seen in *in vitro* studies are supported by animal data. Rats pre-infused with resveratrol prevented reperfusion-induced arrhythmias and mortality [81]. Similar to cardioprotective effects, resveratrol improved insulin sensitivity, glucose tolerance, and lipid profiles in obese animals [82].

One study showed improved flow-mediated dilation (FMD) of the brachial artery when patients with metabolic syndrome consumed 100 mg/d of resveratrol for three months. However this improved blood flow lasted only three months after the study was completed [83]. In another study (84), overweight and obese adults were given a single dose of resveratrol (30 mg, 90 mg, or 270 mg). All three doses improved FMD at 60 min after consumption [84] and in a follow up study, FMD improved in subjects consuming a single dose of resveratrol (75 mg) or daily doses (75 mg) for six months [85]. Subjects consuming resveratrol had significantly reduced VCAM [86], reduced circulating inflammatory markers, C-reactive protein, tumor necrosis factor, and plasminogen activator inhibitor-1 [87] reduced oxidized LDL and apolipoprotein B [88].

Resveratrol consumption (1000 mg/d for 45 days) significantly lowered fasting glucose, insulin concentrations, hemoglobin A-1c levels, and insulin sensitivity (HOMA-IR) in subjects with type 2 diabetes [89]. Similar glycemic benefits were observed at a lower dose of 250 mg/d for three months [90], and at a very low dose of 10 mg/d for four weeks [91]. These data show that resveratrol consumption could improve cardiovascular health as well as type 2 diabetes control.

### 3.3. Lignans

Lignans are polyphenols found in plants, especially in flaxseed (secoisolariciresinol diglucoside), sesame seeds (sesamin, sesamolin), and soy, followed by whole-grains cereals (syringaresinol), and legumes, including nuts. Flaxseed contains approximately 335 mg lignans/100 g [92] and sesame seeds contain approximately 373 mg/100 g [93]. Fruits and vegetables contain a wide variety of lignans (e.g., matairesinol (MAT), pinoresinol (PINO) and lariciresinol (LARI)) but in minute quantities [94].

Consumption of lignans is associated with decreased risk of CVD. The proposed mechanisms by which dietary lignans could reduce the risk of CVD include the phytoestrogenic, and antioxidant activity of these compounds and their metabolites. First, some plant lignans such as matairesinol (MAT), secoisolariciresinol (SECO), pinoresinol (PINO), and lariciresinol (LARI) are metabolized by intestinal bacteria to enterolignans (enterodiol and enterolactone) in various proportions [95,96,97], absorbed, and transported into systemic circulation. Enterolignans, bind to estrogen receptors alpha and beta, which are found in the endothelium of blood vessels and exert weak estrogenic activity [98]. Additionally, they promote the synthesis of sex hormone-binding globulin and inhibit the activity of several enzymes including aromatase and 5a-reductase [99]. The second is associated with the antioxidant activity of lignans and their metabolites [100]. In middle age–elderly men and post-menopausal women living in Northern Italy SECO, LARI, and total lignan intake decreased plasma soluble intercellular adhesion molecule-1 (sICAM-1). Soluble intercellular adhesion molecule-1 is the circulating form of ICAM-1 that is expressed on cell surface of many cell lines including endothelial cells. Interaction between ICAM-1 and lymphocyte function-associated antigen LFA-1 increases leukocyte adhesion and migration across the endothelium. Both ICAM-1 and sICAM increase the risk of atherosclerosis. In the same study, MAT intake increased FMD, another measurement of the endothelial function of blood vessels providing further evidence that plants contain bioactive compounds which reduce the risk of CVD [101].

The next section describes in detail three unrelated groups of food bioactives with potentials as agents for the management of CVD and diabetes. These include resistant starch—a carbohydrate, and two classes of small molecules—one derived as a result of food processing/manufacturing and the other endogenous to fruit berries.

## 4. Select Examples of Bioactive Plant Metabolites with Potentials for Management of Non-Communicable Diseases

### 4.1. Resistant Starch

Complex carbohydrates derived from starch contribute over half of humans’ daily energy requirements. Starch is a homopolysaccharide made in plants and stored in granules. Amylose and amylopectin are two polymers found in starch and are identified based on the glycosidic bond linking the α-D-glucose monomers. Amylose is a linear polymer with α-(1,4) linkages while amylopectin has linear α-(1,4) linkages and α-(1,6) branch points.

Starch was once considered to be completely digestible because human enzymes could cleave the amylose and amylopectin linkages entirely in the small intestine. However, in 1982, Englyst discovered that some foods contain small amounts of starch that resist enzymatic hydrolysis [102]. This starch is known as resistant starch (RS) and escapes digestion in the small intestine entering the large intestine intact. Four types of RS (RS 1 through RS 4) were classified by Englyst ten years after its discovery, which are based on its physical, structural, or chemically-modified properties [103]. Dietary sources of RS 1 include partially milled grains and seeds. RS 2 can be found in raw potatoes, legumes, just-ripe bananas, and high-amylose maize (HAM). HAM is a functional ingredient containing up to 60% amylose produced by corn breeding technology and is utilized most often in research protocols. RS 3 results from retrograded foods, such as potatoes, cereals, and breads. Chemically- or physically-modified starch and resistant maltodextrins are known as RS 4 and 5, respectively. The average daily global consumption of RS ranges from 3 to 10 g [104]. RS is considered both a dietary and functional fiber, thus increasing consumption of RS could improve fiber intake and overall health [104,105].

The digestive properties of RS in the small and large intestine, or lack thereof, contribute to its bioactivity and subsequent metabolic outcomes. Due to lack of enzymatic hydrolysis, the direct contribution of glucose to blood from RS is minimal and allows for an attenuated post-prandial glycemic response. The acute blood glucose response can be specific to RS type, where RS4 can be more effective than RS2; however, consuming RS4 over 12 weeks did not significantly lower fasting blood glucose [106,107]. Although studies reporting lower glucose concentrations immediately following RS intake have similar amounts of available carbohydrate with the control, results are more pronounced when RS replaces a portion of fully digestible carbohydrate [108,109]. The reductions in blood glucose are also more apparent among individuals with metabolic syndrome or type 2 diabetes who consumed 40 g RS2 daily over a longer duration (8–12 weeks) [110,111]. Of more importance, peripheral insulin sensitivity (Si) also improved by approximately 20% in individuals with metabolic syndrome consuming the same amount or RS that elicited reductions in blood glucose [110,111]. Interestingly, a 33% improvement in peripheral Si resulted without concomitant changes in blood glucose after a meal tolerance test after healthy adults consumed 30 g RS2 over four weeks [112]. Improvements in Si have also resulted with lower amounts of RS2 intake. One study showed a 56.5% improvement in Si in men, but not women, after 15 g of RS2 were consumed daily for four weeks [113]. The women in this study had 26% higher Si at baseline than men, suggesting that improvements in Si may be more apparent among individuals who are insulin resistant. To date, only one long-term study examined Si in adults with well-controlled type 2 diabetes. In this study peripheral or hepatic SI did not improve but changes in blood glucose were observed after daily intake of 40 grams of RS2 for 12 weeks [114]. While RS2 intake improves glucose control and Si, especially among individuals with obesity-related metabolic disorders, the type, amount, and duration of RS consumption must be considered when examining the metabolic impact.

The production of short chain fatty acids (SCFA) from RS fermentation by gut microbiota in the large intestine further classifies it as a bioactive compound. The SCFA are capable of influencing risk, and even treatment, of NCDs such as diabetes and cancer through several mechanisms: decreasing luminal pH, enhancing mineral absorption, and stimulating the release of two satiety peptides known as glucagon-like peptide -1 (GLP-1) and peptide tyrosine tyrosine (PYY) to the periphery [115,116]. GLP-1 is an incretin known to reduce blood glucose concentrations, control gut motility, and influence food intake [117]. The PYY isoform also influences appetite by binding to Y2 receptors in the hypothalamus, thus activating pro-opiomelanocortin neurons that correspond to increases energy expenditure [118]. Interestingly, several animal studies have observed significant increases in GLP-1 and PYY that correlate with reductions in total body adiposity and visceral fat after RS2 intake over time [119]. The mechanism of reduced adiposity may be related to enhanced fat oxidation and reduced lipogenesis [120,121] and increased uncoupling protein-1 in brown adipose tissue [122].

Unfortunately, the favorable changes in body composition and satiety peptides observed in animal models have not been replicated in human studies [123]. Several reasons why animals showed pronounced improvements in satiety peptides and body composition after RS intake than humans include the amount consumed, gut microbiota composition, differences in cecum size and physiology, and age at intervention [124]. RS can act as a prebiotic to selectively increase the concentration and viability of certain bacteria, such as *Ruminococcus bromii* [125]. However, several factors may influence the fermentative capabilities of microbiota, including diet and genetics [125]. In addition, it would be physically impossible for humans to consume the amount of RS administered to animals.

One human study reported enhanced post-prandial GLP-1 response in individuals with type 2 diabetes consuming 40 g RS2 daily for 12 weeks but concomitant changes in glucose or insulin metabolism or body composition were not observed [114,126]. Theoretically, elevated concentrations of GLP-1 should translate to decreased energy intake through the incretin effect and improvements in satiation. Intra-individual variation in gut microbiota may influence RS fermentation, the production of SCFA, and upregulation of GLP-1 [109]. Since the half-life of GLP-1 is 1–2 min, very high concentrations may be required to promote satiety [127]. RS metabolism is also highly variable ranging from 82% to 53% among healthy and hyperinsulinemic adults, respectively [128], suggesting some individuals may excrete a large portion of unmetabolized RS.

The risk of colon cancer and chronic kidney disease may also be influenced by dietary RS. The consumption of 40 g of butyrylated RS2 with red meat (300 g) decreased O6-methyl-2-deoxyguanosie adducts, improved SCFA excretion, and altered the microbiota profile after four weeks [129]. The attenuation of chronic kidney disease progression has also been demonstrated by improving creatinine clearance and reducing inflammation in rats after consuming RS2 [130].

In conclusion, RS is a bioactive, fermentable fiber that can assist with the prevention and improvement of NCDs including insulin resistance and cancer. More research is needed to determine the exact mechanism(s), amount, type, and duration of RS intake, and type of microbiota capable of fermenting RS, to produce optimal metabolic outcomes.

### 4.2. Cyclic Dipeptides

Cyclic dipeptides (also known as 2,5dioxopiperazines; 2,5-diketopiperazines; cyclo (dipeptides); or dipeptide anhydrides) are relatively simple compounds and, therefore, are among the most common peptide derivatives found in nature. Curtius and Gloebel synthesized the first cyclic dipeptide, cyclo (Gly-Gly), in 1888 [131]; however, their existence as a special group of compounds in nature was not recognized until early in the 20th century [132]. Between the late 1800s and early 1900s, many simple diketopiperazines such as cyclo (Gly-Gly) were synthesized for the sole purpose of examining their interesting physicochemical properties [132]. In later years, a variety of dipeptide diketopiperazines were shown to exist in protein and polypeptide hydrolysates as well as fermentation broths and cultures of yeast, lichens, and fungi [133]. Some of these diketopiperazines were thought to result from nonenzymatic cyclization of dipeptides and their amides, inasmuch as they are often formed during chemical and thermal manipulations, as well as during storage of peptides and proteins [134]. Consistent with a role for fermentation process in synthesis of cyclic dipeptides is the observation of high levels of cyclo (His-Pro) in foods that undergo fermentation and/or high heat treatment of protein-rich foods. Such examples are nutritional supplements (e.g., TwoCal HN and Jevity), milk, yogurt, sauces, and fermented fish [135,136,137].

Although cyclic dipeptides are ubiquitous in nature, only very few of these have so far been shown to possess any biological activity. These include, cyclo (His-Pro), cyclo (Leu-Gly), cyclo (Tyr-Arg), and cyclo (Asp-Pro). Of these only cyclo (his-Pro) has been shown to be endogenous to animal kingdom. The demonstration of cyclo (His-Pro) synthesis in mammals was an accidental observation. In 1976, while studying thyrotropin-releasing hormone (pGlu-His-ProNH_2_, TRH) metabolism, we observed that *in vitro* incubation of [^3^H-Pro]TRH with hamster hypothalamic extracts or intraventricular administration of [^3^H-Pro]TRH into rat brain led to the formation of a new metabolite that was characterized to be cyclo (His-Pro). The pleiotropic nature of the biologic activities associated with its precursor TRH, and prevailing acceptance of the role of limited proteolysis in generating new biologically active peptides aroused interest in cyclo (His-Pro). Two major advances—development of specific radioimmunoassay for cyclo (His-Pro) [138] and commercial availability of large quantities of synthetic peptide—helped research in pharmacology of cyclo (His-Pro) and demonstration of its presence in biologic samples.

The research essentially progressed on two fronts—measurement and characterization of CHP in a variety of biological samples including food and evaluation of its potential biologic activities. The data presented in Table 2 summarizes data on measurement of CHP in a number of food products. To date CHP has been shown to exhibit a score of diverse biologic activities that have been reviewed in detail elsewhere [133,134,139,140,141]. Based on the prevailing data in the literature we feel that CHP may be a good potential candidate food bioactive for the management of insulin resistance. Therefore, for the purpose of this review we are going to focus on the role of CHP in insulin physiology in relation to diabetes. Since food intake is an important signal for peripheral excursions in circulating insulin and glucose, we will also review role of CHP in food intake where appropriate.

### 4.3. Cyclo (His-Pro), Food Intake, and Body Weight

Morley *et al.*, (1981) were the first to demonstrate that intracerebral administration of CHP to rats led to suppression of food intake induced by stress, fasting, or spontaneous feeding [142]. While many investigators [143,144,145] have supported this initial observation, Bowden *et al.*, (1988) could not demonstrate any anorectic role for CHP [146]. An anorectic or satiety-modulating role for CHP is supported by elevation of CHP in brains of hyperphagic obese Zucker rats [147] and fasted Sprague–Dawley rats [148] and changes in plasma CHP in patients (anorexia nervosa and bulimia) with satiety disturbances [149].

These initial observations supporting an appetite suppressant and satiety-inducing role of CHP led to further examination of its role in body weight control. Treatment of aged (16–18 months), obese, and insulin-resistant Sprague–Dawley rats with diet rich in CHP for eight weeks resulted into improvement of oral glucose tolerance and body weight control [150]. In a follow-up study, using genetically type 2 diabetic Goto–Kakizaki (G-K) rats and aged obese Sprague–Dawley (S-D) rats, these investigators made a similar observation following treatment with synthetic CHP and zinc combination [151].

### 4.4. Cyclo (His-Pro), Insulin Secretion, Glucose Metabolism, and Diabetes

The demonstration of appetite inhibitory function of CHP and changes in its level with feeding-fasting coupled with availability of specific radioimmunoassay of CHP, led us to explore its role in insulin physiology. In 1983, we demonstrated presence of CHP in rat pancreatic islet at a level 88-fold higher than whole pancreas [152]. Soon after this observation, Leduque *et al.*, (1987) using immunohistochemical techniques demonstrated localization of CHP within pancreatic alpha cells that also harbor glucagon/glicentin [153]. CHP may act here like an incretin in augmenting insulin secretion and, thus, reducing plasma glucose. Since CHP is localized in alpha and not beta-cells of the pancreas, it may act upon beta cells to stimulate insulin release via a paracrine mechanism. A further support for a possible role of CHP in glucose metabolism comes from a very rapid rise (maximum at 15 min) in blood CHP during oral glucose tolerance test (OGTT) in normal human subjects [154]. In support that CHP may act like an incretin of gut origin, Hilton *et al.*, (1990) observed an acute and reversible rise in plasma CHP after glucose ingestion in rats and the response was greater after oral glucose than after intravenous glucose [155].

Two lines of investigation support a possible role of CHP in insulin secretion and glucose metabolism. First, in a series of animal experiments, two groups of investigators independently demonstrated improvement in insulin sensitivity following consumption of raw vegetables [156] or spent brewer’s yeast hydrolysate [157] diets both rich in CHP. Second, since plasma levels of CHP are altered by oral glucose ingestion [154], Prasad and co-workers wondered whether exogenous CHP might alter the insulin response to oral glucose ingestion. To this end, rats were given 3 g/kg oral glucose load with either saline or increasing doses of CHP and plasma levels of insulin, C-peptide, and glucose were measured. The results showed CHP to cause higher insulin excursions without any change in C-peptide suggesting that CHP may decrease hepatic insulin clearance. An examination of the relationships between the levels of CHP in obese women and parameters of insulin secretion (fasting C peptide/insulin molar ratio) further suggested a role for this peptide in HIC and hyperinsulinemia in obesity [158].

**Table 2:** Presence of cyclo (His-Pro) in some common foods. Nutritional supplements were products of Ross Laboratories, Columbus, OH (1–8), Norwich Eaton, Norwich, New Your (9), and Travenol Laboratories, Deerfield, IL (10 and 11). Protein source: ^1^ Casein + Soy, ^2^ Casein, ^3^ Essential amino acids, ^4^ Crystalline amino acids. ND = Not detected.

Food	Cyclo (His-Pro) Concentration	Reference
Noodle	18.8 ng/g	[136]
Potted Meat	40.9 ng/g	[136]
Nondairy creamer	30.0 ng/g	[136]
Hot Dog	18.1 ng/g	[136]
Ham	32.5 ng/g	[136]
Egg	5.7 ng/g	[136]
White Bread	21.8 ng/g	[136]
Tuna	510.4 ng/g	[136]
Fish Sauce	1291.8 ng/g	[136]
Dried Shrimp	1630.8 ng/g	[136]
Fresh Cow Milk	1.8 ng/g	[135]
Pasteurized Cow Milk	2.5 ng/g	[135]
Yogurt	4.2 ng/g	[135]
Spent Brewer’s Yeast hydrolysate	674,000 ng/g	[157]
**Nutritional Supplements**		
(1) Ensure plus ^1^	300 ng/mL	[137]
(2) Ensure HN ^1^	601 ng/mL	
(3) Ensure ^1^	454 ng/mL	
(4) Pulmocare ^2^	824 ng/mL	
(5) Enrich ^1^	323 ng/mL	
(6) TwoCal HN ^2^	4763 ng/mL	
(7) Jevity ^1^	4467 ng/mL	
(8) Osmolite ^1^	123 ng/mL	
(9) Tolerex ^3^	ND	
(10) Travasorb Hepatic ^4^	ND	
(11) Travasorb Renal ^4^	ND	

**Figure 1:**
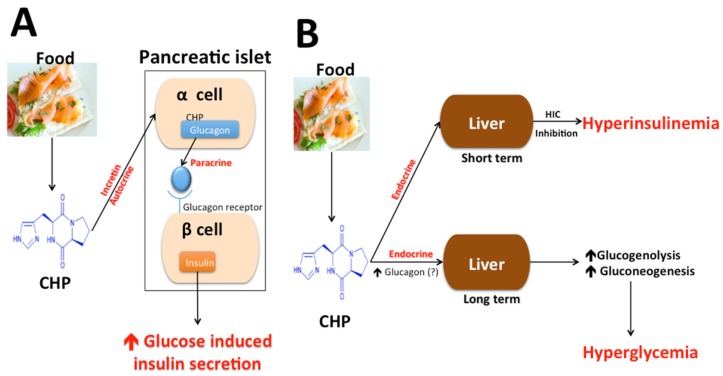
A diagrammatic representation of actions of Cyclo (His-Pro) at the level of the pancreas and liver in controlling insulin secretion and glucose metabolism. CHP = Cyclo (His-Pro). Panel A refers to action of CHP on pancreatic α and β cells via autocrine/incretin mechanisms. Panel B refers to short-term and long-term actions of CHP on liver.

In Figure 1, we have made a graphic attempt to visualize the complex role of CHP in controlling insulin secretion and glucose both in short and long terms at the level of pancreatic alpha and beta cells and liver acting as incretin, as well as autocrine, paracrine, and endocrine mediators. While our assumption of CHP stimulation of glucagon secretion remains to be examined, human beta cells express functional glucagon receptors which can, similar to incretin hormone receptors, generate synergistic signals for glucose-induced insulin secretion [159,160,161].

While the mechanism underlying changes in hepatic insulin clearance is not clearly deciphered, prolonged decrease in HIC has been clearly associated with risks for insulin resistance, dyslipidemia, metabolic syndrome, and CVDs [162,163,164]. Acutely, CHP may help glucose metabolism by enhancing glucose-induced insulin secretion, a benefit to those with insulin resistance.

This opens further possibilities of new lines of investigation into the role of CHP and similar bioactive food components in the etiology of metabolic diseases.

### 4.5. Fruit Berries

Berries and other fruits rich in polyphenols and other natural compounds with bioactive properties have shown promise in recent years for use in the prevention of NCDs such as arthritis, diabetes, cancer, and CVD [165,166,167,168,169]. Antioxidant vitamins found in berries, such as vitamins C and E, may be important contributors to these outcomes [168]. Polyphenols found in berries and other plant foods are particularly associated with anti-inflammatory, antioxidant, cardioprotective, and chemopreventive properties, making them an ideal subject of research for chronic disease applications [167,168,169]. The mechanism of action is not fully understood, but appears to include down-regulation of the inflammatory cytokines, or by antioxidant or anti-inflammatory pathway signaling [166].

### 4.6. Bioactive Berry Constituents

A wide variety of phenolic compounds act as a protective mechanism against injury in the fruit. These compounds contribute to the antioxidant properties of berries and are typically found in the outer parts of the fruit or berry, most often as cinnamic and/or benzoic acid derivatives [166]. Anthocyanins tend to be located in the skin of the fruit, but are less strongly correlated to antioxidant activity than total phenolic content. Tannins contribute to sensory properties, like the tart taste of berries, and act as a stabilizing factor for the anthocyanins present. Small amounts of carotenoids and stilbenes such as resveratrol are present as well. While a well-known stilbene is the resveratrol found in grapes, smaller amounts of resveratrol can be found in cranberries, strawberries, and other berries. The antioxidant activity of total phenolic compounds can vary, however their Trolox Equivalent Antioxidant Capacity (TEAC) and Trolox values have been shown to be three to 30 times that of ascorbic acid and reference standards [166]. There is likely a synergistic activity among the differing bioconstituents present, with polyphenolic compounds accountable for most of the bioactivity [167].

Chokeberry, bilberry, and blackcurrant berries have the highest antioxidant capacity of the different berry fruits (umol Trolox/g fresh weight), and whole fruit extracts have greater antioxidant activity than many isolated phenolic compounds or vitamins [170]. However, more common fruits also contain bioactive compounds that may reduce the risk of chronic disease. Strawberries are known to be high in phenolic compounds such as the phenolic acid derivative ellagic acid, and contain a significant amount of vitamin C. Blueberries are noted for a wide variety of anthocyanin compounds, while both cranberries and blueberries also contain significant concentrations of phenolic acids. The content and antioxidant activity of berry bioactive constituents can be dependent upon plant varietals, and growing, harvest, and storage conditions, as well as processing methods [170]. Since the bioavailability of these compounds differs between individuals [168], there is also a likely genetic component to the effectiveness of berry consumption in preventing NCDs.

### 4.7. Berry Consumption and Non-Communicable Disease

Of all the NCDs, most research regarding berry intake has been conducted in the area of CVD [165,171,172,173,174,175,176,177]. Berries may positively impact a number of preventable risk factors for CVD, including undesirable lipid profiles and hypertension [165,171,173,174,175,176,177,178]. This may be explained via a wide variety of mechanisms, including anti-inflammatory activity, free radical scavenging and up-regulation of antioxidant enzyme genes, decreased levels and antioxidation of LDL, increases in circulating HDL, inhibition of platelet activation and aggregation, and improvements in endothelial function [165,172,176,179,180]. Other observed effects include decreases in hepatic cholesterol formation and fatty acid synthesis, and increases in cholesterol excretion and mitochondrial fatty acid oxidation [181]. Therefore, incorporation of berries and berry products into the diet may represent an accessible and affordable way to decrease cardiovascular risk factors.

Improved plasma antioxidant activity may be due in part to an increased vitamin C intake after regular berry consumption [167]. Del Bo and colleagues detected improvements in oxidative status following a 300 g dose of blueberries, but these were not accompanied by significant changes in peripheral arterial function or NO levels in a group of healthy males [182]. In contrast, a number of studies found increases in antioxidant activity were accompanied by desirable changes in lipid profiles [165,167,181]. Hypercholesterolemic male Dankin Hartley guinea pigs on a blueberry-supplemented diet for 75 days responded with reductions in oxidative stress, serum total cholesterol (TC) and LDL, and hepatic and aortic cholesterol accumulation. However, these observations were only in those animals given a high cholesterol diet. In this high cholesterol group, daily blueberry treatment equal to approximately 3.8–4 g fresh blueberries also decreased serum amino transferases, malondialdehyde (MDA), and diene conjugate (DC), and increased hepatic glutathione transferase (GST) activity when compared to the high cholesterol diet without blueberries [181].

### 4.8. Cardiovascular Diseases

Other promising studies have linked berry consumption to antioxidant activity with accompanying lipid changes in humans [165,167,171,177]. In healthy adult men, two weeks of 7 mL/kg cranberry juice intake resulted in increased antioxidant capacity from baseline (+6.5% ± −10.3, *p* = 0.014) and reduced oxidized LDL (−9.9% ± −17.8, *p* = 0.0131) [177]. In another study, 12 weeks of powdered strawberry beverage consumption was followed by a dose-responsive decrease in serum MDA (a marker of lipid peroxidation) in a group of abdominally obese adults with high serum lipids (low dose: 1.3 ± 0.2 umol/L, high dose: 1.2 ± 0.1 umol/L *vs.* low control: 2.1 ± 0.2 umol/L and high control: 2.3 ± 0.2 umol/L, *p* < 0.05). The low (25 g/d) and high doses (50 g/d) were approximately equal to 250 g and 500 g of fresh strawberries and compared to a control diet matched in fiber and calories. There were also dose-responsive decreases in serum TC, LDL cholesterol, and nuclear magnetic resonance (NMR)-derived small LDL particle concentration, which is associated with atherogenicity. However, adiposity, blood pressure, glycemia and serum HDL, triglycerides, C-reactive protein, and adhesion molecules did not significantly change [165]. In a third study, consumption of 500 g fresh strawberries for one month increased ferric-reducing antioxidant power and oxygen radical absorbance capacity measures in healthy adults (+24.97%, +41.18%, +41.36%, respectively, *p* < 0.05). Serum MDA, urinary 8-OHdG and isoprostanes decreased (31.40%, 29.67%, 27.90%, respectively, *p* < 0.05), as did spontaneous hemolysis, oxidative hemolysis, and activated platelets (*n* = 10, *p* < 0.05). As red blood cell hemolysis can be influenced by membrane lipid peroxidation, these results suggest an antioxidative effect of strawberry consumption. These changes were accompanied by further significant reductions in TC, LDL and triglycerides compared to baseline, (−8.78%, −13.72% and −20.80%, respectively, *p* < 0.05). It is possible the 2 g fiber/100 g fruit (approximately 10 g fiber daily above a normal diet) contributed to the observed decreases in cholesterol in this study [171].

### 4.9. Hypertension

Another benefit of berry consumption prominent in the available literature has been improvements in blood pressure or hypertensive status [183]. In normotensive male Wistar rats, ten weeks of supplementation with 2% (*wt/wt*) freeze-dried blueberry resulted in decreases in systolic blood pressure of 11%–14% compared to the control group. The supplement dosage correlates to approximately 450 g daily of freeze-dried blueberry intake in humans. Improvements were also seen in vascular reactivity, but not mean body weight or lipid profile [172]. Hypertension in rats is linked to impaired NO-dependent endothelial function, therefore the observed effects may be due to increased NO bioavailability via activation of endothelial NO synthase. Circulating polyphenol metabolites have also been shown to inhibit NADPH oxidase, which would improve the bioavailability of NO and, thus, also improve endothelial-dependent vasodilation [172].

Desirable changes in blood pressure have been also demonstrated after berry consumption in adults with cardiovascular risk factors [167,175,176,184]. In a group of 72 older subjects (*n* = 46 women, mean age 58) with one or more risk factors, an average daily intake of 837 mg mixed berry products resulted in modestly decreased systolic blood pressure (SBP) compared to an increase in the control group (−1.5 mm Hg *vs.* +0.5 mm Hg). While diastolic blood (DBP) pressure remained unchanged with treatment, it increased somewhat in the control group (+0.9 mm Hg). A subgroup of participants with baseline high blood pressure demonstrated even greater effects, with a mean decrease of 7.3 mm Hg in the treatment group compared to 0.22 mm Hg in the control (*p* = 0.024). Serum HDL also increased slightly in the berry group compared to control group (5.2% *vs.* 0.6%) [175]. In post-menopausal women with pre- and stage-one hypertension (*n* = 24 per group), daily consumption of 22 g freeze-dried blueberry powder (equal to about one cup fresh blueberries) resulted in decreased SBP and DBP compared to the control group (131 ± 17 mm Hg, *p* < 0.05 and 75 ± 9 mm Hg, *p* < 0.01, respectively). After eight weeks mean SBP was still considered pre-hypertensive, despite the decrease. Brachial-ankle pulse wave velocity, the gold standard marker for arterial damage and predictive of CVD risk, was also lower at the final visit with blueberry treatment (1401 ± 122 cm/s, *p* < 0.01). In addition, NO levels in the blueberry group were higher compared to baseline (9.11 ± 7.95 umol/L), but this was not the case in the control group [184]. In sedentary men and women without cardiovascular risk factors, consuming 250 g freeze-dried blueberry powder daily resulted in decreases in augmentation index (Alx) and aortic systolic pressure (ASP; *p* = 0.24 and *p* = 0.46, respectively) after six weeks of treatment. In addition, a subset of pre-hypertensive patients (n = 9) demonstrated significant reductions in DBP (*p* = 0.38) [185]. ASP is linked to increased CVD risk, and decreased Alx may be related to the decrease in aortic pressure. Interestingly, in a separate shorter study using the same dose in smokers there were no changes in blood pressure [186]. These results suggest that some cardiovascular risk factors, such as elevated blood pressure could be attenuated with berry intake, perhaps via increased NO production which mediates vasodilation.

### 4.10. Diabetes Mellitus

Other studies have demonstrated cardiovascular benefits after berry consumption in adults with diabetes or the metabolic syndrome [174,187,188] *In vitro* studies suggest that anthocyanins may influence gene expression in adipose tissue, which could partially account for a mechanism [189,190]. Six weeks of a 50 g daily strawberry beverage supplement in middle-aged adults with type 2 diabetes (mean age 51.57 ± 10 years) resulted in significantly decreased TC and TC:HDL ratio compared to baseline, but not compared to control (−13.8% and −7.1%, respectively, *p* < 0.00). SBP was also decreased in both treatment groups, but no significant difference existed between groups. However, the strawberry group did have significantly reduced DBP compared to placebo (78.7 ± 7.2 *vs.* 84.4 ± 5.8, *p* = 0.01), and within the strawberry group (84.2 ± 8.03 to 78.7 ± 7.2, *p* = 0.00) [188]. Six weeks of treatment with a twice-daily blueberry smoothie (total 45 g/d, equal to about 2 cups of fresh blueberries) in middle aged adults with the metabolic syndrome resulted in altered endothelial function despite having no effect on blood pressure or insulin sensitivity. Mean resting endothelial function (reactive hyperemia index or RHI) was significantly improved compared to the control group (*p* = 0.024), which persisted after adjustment for confounding factors (*p* = 0.0023). While not significant, 73% of the blueberry group experienced improved endothelial function and 61% of the placebo group experienced decreased endothelial function. It is possible that the absence of change in blood pressure may have been related to antihypertensive medications used by participants, or the more thorough 24-hour ambulatory pressure monitoring. In contrast, no significant changes in peripheral arterial function or NO levels were observed after blueberry intake in adults with the metabolic syndrome [187]. However in an earlier study these researchers did observe improved insulin sensitivity in obese adults [191], an effect also seen in mice fed a high-fat diet [192]. Another study in middle-aged obese men and women with metabolic syndrome used a similar (50 g) dose of freeze-dried blueberry powder, which resulted in SBP lowered by 6% and DBP lowered by 4% compared to the control group (−1.5%, −1.2%) [174].

While the initial literature on the benefits of berry consumption is promising, it is important consider berry types that have established availability and affordability for common use as fresh produce is seasonal and often expensive. However, if consumption of fruits such as berries can be definitively linked to prevention of non-communicable disease, it may be a bargain compared to the cost and side effects associated with conventional treatment. Berries are widely available in fresh or processed forms, but their polyphenolic constituents are more bioavailable in their naturally-occurring form than in supplemental forms [191,192]. Currently the available literature does not allow for definitive recommendations for berry type, dosage or form for a given disease state. Future studies should investigate biomarkers of polyphenol metabolites, and continue to study the effects of whole berry intake in humans with chronic diseases. Despite these limitations, berries are poised to become a prime functional food in the fight against NCDs.

## 5. Future Directions

The use of pharmacologic means for the management of NCDs, such as CVD and diabetes, has played a monumental role in decreasing mortality rate if not morbidity. These pharmacologic agents are generally designed to mimic receptor agonist/antagonist, enzyme inhibitors/stimulators, or supply missing substrates. Since most receptors or enzymes are ubiquitous to many organs and cell types, it is not surprising that these agents bring with them a plethora of side effects. We must emphasize here that use of pharmacologic agents in disease management is of utmost importance. However, considering side effects of treatment, there is an urgent need of search of preventative measures that may delay or abrogate use of pharmacological agents altogether.

Food bioactives are steadily appearing to be in a position to play this preventive role. However, there remain many technological issues in the way of bringing bioactives from bench to bedside. Below is a list of four major issues associated with the development of food bioactives for disease prevention. These include, (i) what is the nature of food bioactives in functional foods; (ii) how to establish limit of maximum purity without losing functionality of food bioactives; (iii) how to establish limit of maximum purity without losing shelf life of the food bioactives; and (iv) how to deliver efficiently and maximize half-life *in vivo*.

Food bioactives include a large class of molecules such as polyphenols, non-fermentable carbohydrates, w-3 fatty acid, beta-carotene, and sulfur-rich compounds, to name a few. To date >5000 phytochemicals have been identified as polyphenols while many more still remain unknown [193] and identified before their health benefits are fully understood. For example, risk of cancer is inversely related to the consumption of green and yellow vegetables and fruit. Since β-carotene is present abundantly in these vegetables and fruit, role of pure β-carotene in cancer prevention has been investigated extensively. However, the role of carotenoids as anticancer supplements has been questioned as a result of several clinical studies [194]. Such observations are not limited to β-carotene only. The reason for such disappointing results may be many. For example, to elicit its anti-cancer properties, β-carotene may need a congener or another food bioactive also endogenous to fruit and vegetables. Fruits and vegetables may have chemical components much needed to maintain bioavailability of β-carotene.

A better understanding of the nature of food bioactives, and their combinations and mechanism of their action in eliciting bioactivity, preservation of their bioactivity in storage, and a better understanding of how to increase their bioavailabilty is needed before effective utilization of food bioactives for disease prevention is achieved. To help bring food bioactives at the center stage of prevention, research must be a cooperative effort between all with expertise in nutrition, food science, analytical chemistry, separation science, pharmacology, and nanotechnology.
